# Study of the Few-Shot Learning for ECG Classification Based on the PTB-XL Dataset

**DOI:** 10.3390/s22030904

**Published:** 2022-01-25

**Authors:** Krzysztof Pałczyński, Sandra Śmigiel, Damian Ledziński, Sławomir Bujnowski

**Affiliations:** 1 Faculty of Telecommunications, Computer Science and Electrical Engineering, Bydgoszcz University of Science and Technology, 85-796 Bydgoszcz, Poland; krzysztof@palczynski.com.pl (K.P.); damian.ledzinski@pbs.edu.pl (D.L.); slawomir.bujnowski@pbs.edu.pl (S.B.); 2 Faculty of Mechanical Engineering, Bydgoszcz University of Science and Technology, 85-796 Bydgoszcz, Poland

**Keywords:** ECG signal processing, few-shot learning, R wave detection, distance-based classification, PTB-XL dataset, deep learning

## Abstract

The electrocardiogram (ECG) is considered a fundamental of cardiology. The ECG consists of P, QRS, and T waves. Information provided from the signal based on the intervals and amplitudes of these waves is associated with various heart diseases. The first step in isolating the features of an ECG begins with the accurate detection of the R-peaks in the QRS complex. The database was based on the PTB-XL database, and the signals from Lead I–XII were analyzed. This research focuses on determining the Few-Shot Learning (FSL) applicability for ECG signal proximity-based classification. The study was conducted by training Deep Convolutional Neural Networks to recognize 2, 5, and 20 different heart disease classes. The results of the FSL network were compared with the evaluation score of the neural network performing softmax-based classification. The neural network proposed for this task interprets a set of QRS complexes extracted from ECG signals. The FSL network proved to have higher accuracy in classifying healthy/sick patients ranging from 93.2% to 89.2% than the softmax-based classification network, which achieved 90.5–89.2% accuracy. The proposed network also achieved better results in classifying five different disease classes than softmax-based counterparts with an accuracy of 80.2–77.9% as opposed to 77.1% to 75.1%. In addition, the method of R-peaks labeling and QRS complexes extraction has been implemented. This procedure converts a 12-lead signal into a set of R waves by using the detection algorithms and the k-mean algorithm.

## 1. Introduction

Machine learning, especially Deep Learning (DL) approaches, has been of interest in academia and industry. This has resulted in numerous changes in the approach to automatic detection or classification processes. However, the reliability of such studies has not always been high and differs depending on the methods used.

Since recently, it has been proved that Artificial Intelligence (AI) and machine learning has numerous applications in all engineering fields. Among them are the areas of electrical engineering [1], civil engineering [2], and petroleum engineering [3]. In addition, classification using DL methods [4] have several practical applications in various areas of medicine, such as the diagnosis of diseases based on physiological parameters [5], the classification of cardiac arrhythmias based on ECG signals [6,7], and the recognition of human activity [8]. Various ECG classification schemes based on DL were used to detect heart diseases [9,10,11,12], for example, using Long Short-Term Memory networks [13] and one-dimensional Convolution Neural Networks [14,15,16]. In addition, DL methods have been used to classify pathological conditions of the heart, such as arrhythmia, atrial fibrillation, ventricular fibrillation, and others.

**Figure 1:**
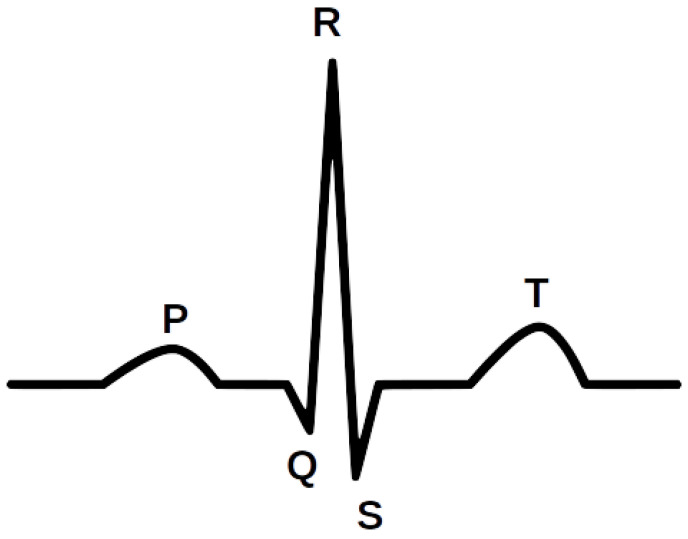
The illustrative waveform of the ECG signal.

Cardiovascular disease is a general term for a series of cardiovascular abnormalities that are the world’s leading cause of death [17]. Each of them is identified and interpreted using an electrocardiogram (ECG). The ECG is an important non-invasive diagnostic method for the interpretation and identification of various types of heart disease. Figure 1 shows an illustrative waveform of the ECG signal. Every day, approximately 3 million ECGs are produced worldwide [18]. ECG data contain rich information about the rate and rhythm of the heartbeat. Clinically, the ECG is analyzed over a short period using a graph of several consecutive cardiac cycles. The process begins with R-peak detection. It is usually the most visible part of the ECG that can be easily identified. The ECG reflects the depolarization of the main mass of the ventricles and refers to the maximum amplitude in the QRS complex. QRS complexes are the starting point for the analysis of the ECG signal. They serve as rhythm items and provide information about intraventricular rhythm and conduction [19,20].

Several methods and techniques have been used to locate the R-peak in the ECG signal, based on standard techniques such as digital filtering, wavelet transform, Fourier Transform, signal decomposition, and Hilbert Transform. However, only a few proposed works use DL methods in the literature to detect QRS complexes. One of the works in [21] is where a 300-point Convolutional Neural Network (CNN) and clustering on the neural output are used to detect QRS complexes on the pre-processed input signal. Another method using CNN has been proposed [22], demonstrating the reliable detection of the fetal QRS complex. The authors of the work [23] proposed a 1-D CNN and Multi-Layer Perceptron (MLP) classifier that determines the QRS positions. Another approach was the work [19] in which two DL models based on multi-dilated convolutional blocks were used: CNN and CRNN. Finally, this group of works includes [24], where a stacked autoencoder deep neural network is proposed to extract the QRS complex.

Regardless of the DL methods chosen, problems are identified, including classification efficiency, the detection of undesirable results, dependence on computing power, and the high sample count. In response to these problems, a few newly published articles propose using Few-Shot Learning (FSL) to identify new concepts in medicine and fill the gap between the efficiency and the size of the training samples. FSL mimics humans’ ability to acquire knowledge from a few samples. This technique involves training a neural network to encode input data into small-sized vectors, which distances to other vectors encoding objects of the same class are smaller than to vectors representing objects from different classes. The distance between vectors is usually computed by measuring the Euclidean distance between two vectors. In addition, FSL can encode information regarding the object’s belonging to a particular class in the output vector. Because of that, the layer of neurons representing defined classes is not required, which allows the FSL network to distinguish between classes that were not seen during training, thereby enabling learning from limited samples and rapidly generalizing to new tasks, giving a different perspective on DL.

There are many areas of application of FSL methods. In the medical field, the use of FSL methods occurs in conjunction with medical images and medical signals. One of the application directions is to use the network-based FSL method to classify rare human peripheral blood leukocyte images. The proposed Siamese network by the authors of [25] contains two identical Convolutional Neural Networks and a logistic regression network. In justifying their research, the authors point to the relationship between the number of leukocytes and various diseases, including cancer. The obtained results show that the Siamese network can overcome the scarcity and imbalance of datasets used in this research. The results are promising and give hope for addressing the issue of rare leukocyte images recognition in medicine.

Another view is the use of Few-Shot Deep Learning in medical imaging, for example, COVID-19-infected areas in Computed Tomography (CT) images. Recent studies indicate that detecting radiographic patterns on chest CT scans can provide high sensitivity and specificity in identifying COVID-19. One of the works [26] was undertaken to investigate the efficacy of FSL in U-Net architectures, allowing for a dynamic fine-tuning of the network weights as new samples are fed into the U-Net. The obtained results confirmed the improvement of the segmentation accuracy improvement in the identification of COVID-19-infected regions. A similar approach was proposed by the authors of another study [27], pointing to the use of FSL for the computerized diagnosis of emergencies due to coronavirus-infected pneumonia on CT images. A similar application of FSL was demonstrated by the authors of the study [28], who undertook the classification of COVID-19 infected areas on X-rays. As part of the research, the method was tested to classify images showing unknown symptoms of COVID-19 in an environment designed to learn several samples, with prior meta-learning only on images of other diseases.

Diagnostics of disease states based on medical images using DL methods have also been applied in dermatology. The authors of the work [29] demonstrated the possibility of using FSL for Dermatological Disease Diagnosis. Skin diseases are increasingly becoming one of the most common human diseases, contributing to dangerous cancerous changes or affecting motor disability. The proposed method is scalable to new classes and can effectively capture intra-class variability. A similar approach was used by the authors of [30], who proposed a Few-Shot segmentation network for skin lesion segmentation, which requires only a few pixel-level annotations. The authors emphasize that the proposed method is a promising framework for Few-Shot segmentation of skin lesions. The conducted experiments show that removing the background region of the query image both accelerates the speed of network convergence and significantly improves the segmentation efficiency.

The works of other authors in medicine with the use of FSL indicate the possibility of application in creating predictive models of drug reactions based on screens of cell lines. For example, the authors’ work in [31] applied Few-Shot machine learning to train a versatile neural network model in cell lines that can be tuned to new contexts using a few additional samples. The model quickly adapted to switching between different tissue types and shifting from cell line models to clinical contexts.

In biomedical signals, an interesting approach is to use the FSL method of Electroencephalography (EEG)-based Motor Imagery (MI) Classification. The authors of the work [32] drew attention to an essential aspect of research on the brain–computer interface using EEG signals. In their justification, they indicated the potential of EEG in designing key technologies in both healthcare and other industries. The research proposed a two-way Few Shot network that can efficiently learn representative features of unseen subject categories and classify them with limited MI EEG data.

In the area of the ECG signal, the authors in [33] proposed a meta-transfer-based FSL method to handle arrhythmia classification with the ECG signal in wearable devices. The results obtained by the authors indicate that the proposed method exceeds the accuracy of other comparative methods when performing various Few Shot tasks within the same training samples.

The study aimed to determine the usefulness of the FSL for ECG signal proximity-based classification. The research was conducted by training Deep Convolutional Neural Networks to recognize 2, 5, and 20 different heart disease classes. For this task, two neural networks were trained. The first one was optimized by performing FSL to classify input samples based on Euclidean distance to the defined classes’ vectors. The second one was trained to perform softmax-based classification. It serves as a basis for comparison due to its well-known effectiveness in recognizing classes established during training. This work also examines classification strategies in FSL by comparing the results obtained from proximity-based classification to training machine learning algorithms on top of optimized FSL neural networks. The tested machine learning algorithms are XGBoost, Random Forest, Decision Tree, K-Nearest Neighbors, and SVMs. The neural network proposed for this task interprets a set of QRS complexes extracted from ECG signals. The method of R-peaks labeling and QRS complexes extraction has been implemented. This procedure converts a 12-lead signal into a set of R waves by using the detection algorithms and the k-mean algorithm. The novelty of this work involves using the FSL learning style for training on known, fixed classes; its comparison with more typical, softmax-based classifications; and the evaluation of classification strategies to be employed on top of the trained FSL network.

This paper is organized as follows: Section 2 closely describes the methods, the architectures of the artificial intelligence system, and the previously carried out data filtering, R Wave detection, and QRS extraction. Then, Section 3 presents the result of the research. Then, the discussion is given in Section 4. Finally, Section 5 concludes the paper and provides a look at further studies on this topic.

## 2. Materials and Methods

**Figure 2:**
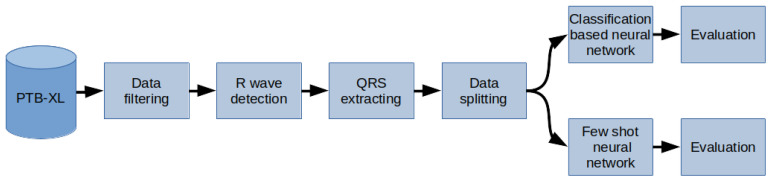
General overview diagram of the method.

The methodology used in the paper was as follows (Figure 2): The PTB-XL dataset containing the labeled 10-second raw-signal ECG was used for the research. First, the records in the database have been filtered. Then, the R waves were labeled in the records in the next step. On this basis, QRS segments were separated. Finally, the dataset has been split into training, test, and validation data (respectively 70%, 15%, 15%). These data were used to train two neural networks, based on softmax and a Few Shot, as classifiers of 2, 5, and 20 classes of heart diseases. In the last stage, the network performance was evaluated.

### 2.1. PTB-XL Dataset

In this study, all the ECG data used come from the PTB-XL dataset [34,35]. PTB-XL is the publicly available and most extensive set of clinical ECG data. It provides a rich set of ECG annotations and additional metadata, which together constitute an ideal source for training and evaluating machine learning algorithms. The PTB-XL dataset contains 12-lead 10 s ECGs from 18,885 different patients for a total of 21,837 records. ECG files come in two other options with 500 Hz and 100 Hz sampling rates with 16-bit resolution. The research used ECGs with 500 Hz sampling rates. The database contains 71 types of heart diseases with 5 significant classes: normal ECG (NORM), myocardial infarction (CD), ST/T change (STTC), conduction disturbance (MI), and hypertrophy (HYP).

### 2.2. Data Filtering

**Figure 3:**
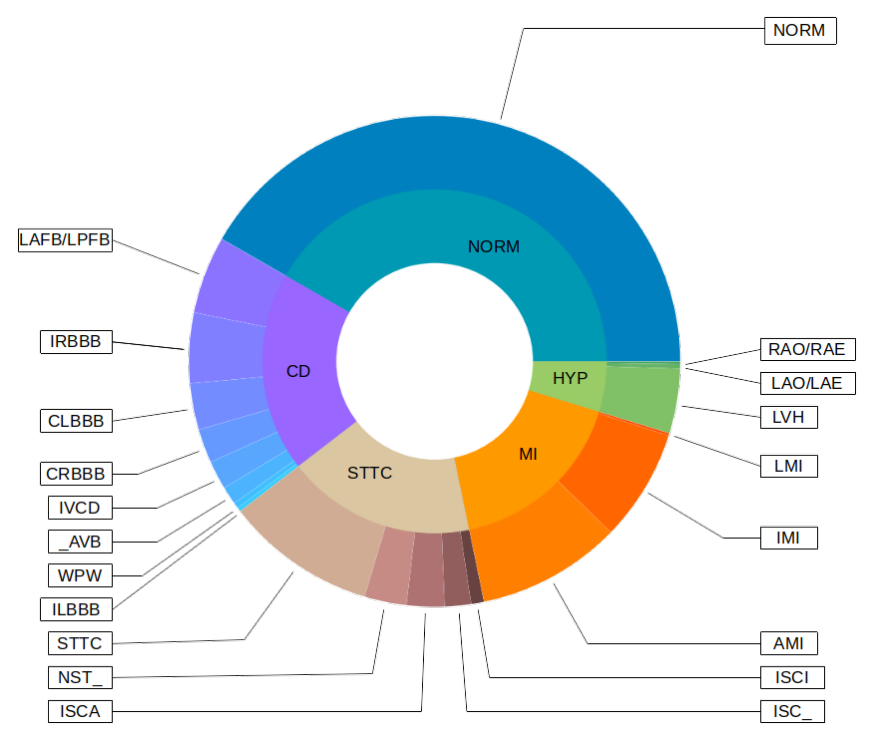
Classes and subclasses of used records.

Initially, the PTB-XL had 21,837 records. However, not all records have labels (assigned classes), and not all assigned classes were 100% sure. For this reason, both cases were filtered out of the original dataset. Each record has a given class and a subclass for specific heart disease. Records with the number of subclasses less than 20 were also filtered from the original dataset. In this way, 17,232 records were obtained, each belonging to 1 of the 5 classes and 1 of the 20 subclasses. Figure 3 shows a detailed distribution of classes and subclasses. Descriptions of the classes of diseases are included in the in Appendix A and Appendix B.

### 2.3. R Wave Detection and QRS Extraction

**Figure 4:**
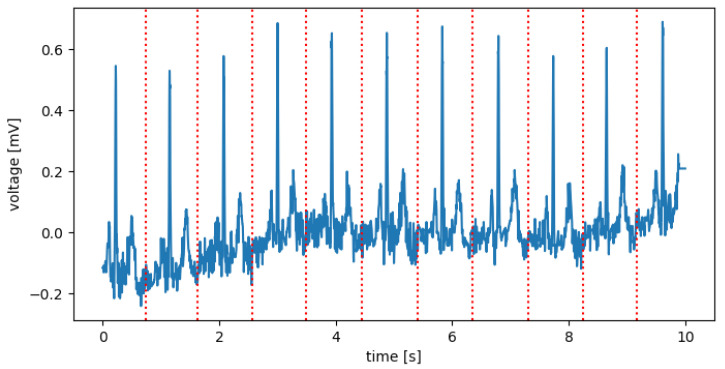
Sample record of NORM class for I lead, with places for section cuts (Red).

None of the known R-peak detection methods tested by the authors were 100% effective. In addition, these methods use only a 1-lead signal. For this reason, the authors decided to propose their own method, using several known methods (Hamilton detector [36], Two Average detector [37], Stationary Wavelet Transform detector [38], Christov detector [39], Pan–Tompkins detector [40], and Engzee detector [41] with modification [42]) for all 12-leads and obtaining a consensus from them using k-mean algorithm. The designated R-peaks were used to cut the 10-s records into segments referred to further in the work as QRS complexes. The cuts were determined in the middle of the distance between the designated R-peaks (Figure 4). The first and last segments were removed. The following segments were resampled to 100 samples. In this way, for each record, a set of QRS complexes and metadata as BPM (Beat Per Minute) and resampling ratio for each QRS complex were obtained.

### 2.4. Designed Network Architectures

**Figure 5:**
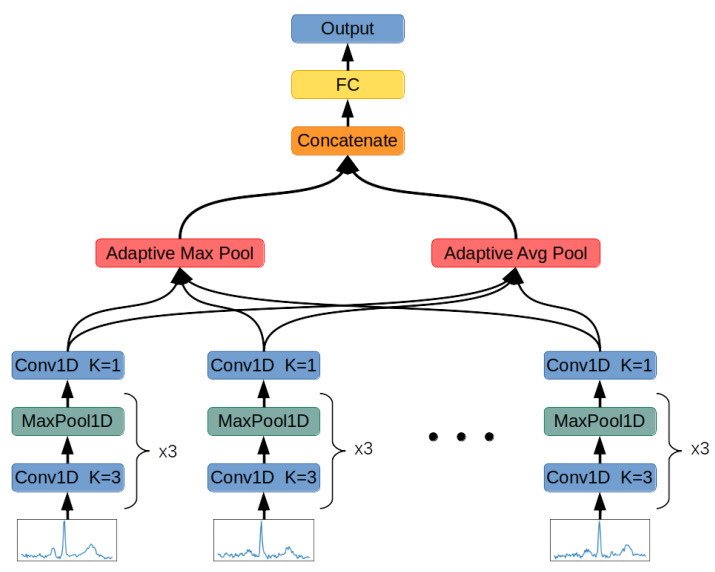
Designed Neural Network architecture.

This chapter describes the architecture of the Deep Neural Networks used in this research (Figure 5) and the methodology of processing QRS complexes, applied loss functions, and training procedure.

The system receives the collection of QRS complexes stored in the input signal:

(1) $$X_{i}=\left\{Q_{1},...,Q_{n}\right\},n\in N^{+}$$

where:

*X*—set of input signals after QRS extraction performed;
*i*—index of signal being processed by the system;
$Q_{n}$—*n*-th extracted QRS complex containing 100 12-dimensional samples:
  (2) $$|Q_{j}|=1200,j\in N^{+}\cap j\leq n$$

Then, a set of QRS complexes is processed by the function designed to transform each wave into a 24-dimensional vector containing abstract features allowing for similarity calculation between vectors representing classes defined in the PTB-XL dataset:

(3) $$f:R^{12\times 100}\to R^{24}$$

The function has been approximated by the deep convolutional neural function described in Table 1. The process of learning this neural network has been presented in the Section 2.5.

Each convolutional layer’s output is subjected to the LeakyReLU activation function with parameter equal to 0.01. The last convolutional layer operates using a kernel of size 1. This computation has been inspired by GoogLeNet architecture [43], and its task is to perform dimensionality reduction. This layer requires only 192 weights to reduce the activation map size 48 times.

The function approximated by Convolutional Neural Network is used to encode each QRS in the input data:

(4) $$Z_{i}=\{f\left(X_{i,j}\right)|j\in N^{+}\cap j<|X_{i}\left|\right\}$$

As a result, $Z_{i}$ is a set of 24-dimensional vectors with varying cardinality. This set is now processed by Adaptive Maximum Pooling and Adaptive Average Pooling functions.

The Adaptive Maximum Pooling function selects maximum value from each dimension of the vectors in the set:

(5) $$Zmax_{i}=[max(\{Z_{i,j,1}\left|j\in N^{+}\cap j<\right|X_{i}|\}),\cdots ,max(\{Z_{i,j,24}\left|j\in N^{+}\cap j<\right|X_{i}\left|\})\right]$$

The Adaptive Average Pooling function averages values of every dimension from vectors in the set:

(6) $$Zavg_{i}=\left[\frac{1}{|Z_{i}|}\sum_{j=1}^{|Z_{i}|}Z_{i,j,1},\cdots ,\frac{1}{|Z_{i}|}\sum_{j=1}^{|Z_{i}|}Z_{i,j,24}\right]$$

The results of both Adaptive Average Pooling and Adaptive Maximum Pooling are combined into 1 48-dimensional vector:

(7) $$A=[Zmax_{i},Zavg_{i}]$$

In the last step, the result is inputted to a fully connected layer with 20 neurons turning the 48-dimensional vector of concatenated pooling results into a 20-dimensional final vector:

(8) $$F_{i}=f\left(A\right);f:R^{48}\to R^{20}$$

Vector $F_{i}$ describes the input signal using 20 abstract features. It is used in both classification neural networks to determine the signal’s class by subjecting it to softmax function for class probability distribution computation or in FSL for signal’s class determination by measuring Euclidean distance to the center of the class represented by vector made of averaging feature vectors obtained from signals on the training dataset. In the case of standard classification, there is also one more fully connected layer added to adjust the size of the abstract features vector to the number of classes in the classification task.

### 2.5. Training

The neural networks’ parameters have been adjusted using Adam [44] optimizer. In addition, the dataset has been split into training, validation, and test sets five times to reduce the impact of fortunate weights randomization on the network’s performance. The split was performed by dividing the dataset by 70%, 15%, and 15%.

The training dataset was used to determine the values of the network’s weights. In addition, the network was evaluated on the validation dataset during the training process to perform early stopping [45] for overfitting reduction purposes. The final network’s evaluation has been performed on a test dataset using the last saved set of weights, which scored the best result on the validation dataset. Each time the network scored the best result on the validation dataset, its weights have been saved. The training lasted until 10,000 epochs elapsed or early stopping was performed.

For the purpose of this research, two neural networks have been trained, one for FSL and one for standard classification serving as a basis for a benchmark. Both networks are structurally almost identical and differ only in adding one fully connected layer in standard classification tasks and the interpretation of output vector and employed loss function.

#### 2.5.1. Few-Shot Learning

Few-Shot Learning network was trained using the triplet margin loss function [46]. The task of this loss function is to decrease the distance between vectors belonging to the same class and increase it for vectors from different classes. This process can be described by the formula:

(9) L(a,p,n)=max(d(a,p)−d(a,n)+m,0)

where:

*a*—“anchor” vector. This vector is compared with the other two vectors;
*p*—“positive” vector. This vector belongs to the same class as the “anchor” vector;
*n*—“negative” vector. This vector belongs to the different class as the “anchor” vector;
*m*—margin. Quantity describing desired separation of vectors from the same class with vectors from different classes. In this research, *m* was equal to 1;
*d*—distance function, $d:\left(R^{20},R^{20}\right)\to R^{1}$.

For this research purpose, the Euclidean distance has been used as a distance function:

(10) $$d\left(x,x^{\prime }\right)=\sum_{j=0}^{\left|x\right|}{\left(x_{j}-x_{j}^{\prime }\right)}^{2}$$

The purpose of the triplet margin loss function is to ensure that the distance between vectors from two different classes is higher than a distance between vectors of the same class in addition to constant margin *m*. The neural network is not penalized for its performance only if:

(11) d(a,p)−d(a,n)+m≤0

(12) d(a,p)≤d(a,n)−m

Minimizing this function ensures the separation of inter-class distances from distances to vectors of other classes by the margin of *m*.

During training, triplets of vectors, two from the same class and one from different classes, were randomly selected and fed to the network. At each step, classes were picked from the distribution created from the computing frequency of occurrence in the dataset. This approach was motivated by the a priori assumption that reciprocating class observation frequency from dataset to training process results in better network convergence. However, for more balanced training, a different approach may be undertaken, in which classes are picked from either a weighted frequency-based distribution or a univariate one.

Due to the PyTorch limitation of forming only homogenous-sized tensors, the process of forming batches requires one more restriction on the triplet sampling function. Every sample in the batch must have the exact number of QRS complexes. The batch-sampling function first randomly selects the number of QRS complexes required in this batch to obtain such tensors. Then, it randomly selects triplets from signals in the dataset that contain the same amount of QRS complexes as the value selected. Finally, the amount of QRS complexes in the batch is sampled from the distribution weighted by the frequency of each wave in the dataset. The evaluation process of the neural network consists of these steps:

1. Split evaluation dataset randomly into two sets while ensuring that QRS complexes for each class have the same cardinality. From now on, the first set is referenced as a “database” set and the second one as a “query” dataset.
2. Use an Artificial Intelligence system to convert each set of QRS complexes from both “database” and “query” datasets into 20-dimensional vectors.
3. For each class, take all vectors belonging to it from the “database” set and compute the average 20-dimensional vector. It results in average vectors being later referenced as “class center vectors”.
4. For each vector in the “query” dataset, compute its distance to every “class center vector”. The class, whose “center vector” has been the closest to the vector from the “query” dataset is the class associated with the entry in the “query” dataset.
5. Calculate evaluation metrics by comparing true labels of vectors in the “query” dataset with labels computed in the previous step.

This process emulates the behavior of the real-life working environment. The “database” set resembles the structure that stores previously measured and processed ECG signals labeled by professionals. This database is used to label incoming queries. In this research, entries in the database were aggregated by computing the average for each class. This solution involves the least amount of computational cost. It is because “class center vectors” are computed once. Then, the incoming query must be compared with only one vector per class instead of numerous database entries, as required in other strategies.

The other method of classification involved training machine learning models on top of network-encoded small-sized vectors. The machine learning models evaluated in this work are XGBoost, Random Forest, Decision Tree, K-Nearest Neighbors, and SVMs with linear, polynomial, radial basis function, and sigmoid kernels. In this approach, the FSL neural network generates small-size vectors encoding crucial features of the input signals. Then, the aforementioned machine learning algorithms are trained to classify these vectors.

#### 2.5.2. Softmax-Based Classification

Softmax-based classification is a well-known process of training a neural network using the operation mentioned above as an activation function for converting the neural network’s output into a class probability distribution. The equation of the softmax function is given below:

(13) $$\sigma {\left(Z\right)}_{i}=\frac{e^{Z_{i}}}{\sum_{j=1}^{\left|Z\right|}e^{Z_{j}}}$$

where:

*Z*—output vector computed by neural network;
$\sigma {\left(Z\right)}_{i}$—value of class probability distribution function for *i*-th class.

The output of the softmax activation function is then compared with the desired results using cross-entropy loss function computed with the formula below:

(14) $$L\left(p,y\right)=-\sum_{c=1}^{M}y_{o,c},ln\left(p_{o,c}\right)$$

where:

*p*—probability that observation *o* belongs to the class *c* computed by application of softmax function on the output of neural network; *y*—binary value that is equal to 1 if observation *o* belongs to the class *c* and 0 if not.

The loss function forces the neural network to output the vector as close as possible to a one-hot encoded vector with the maximum value contained under the index of the class the signal belongs to. This is a well-established solution tested both by scientists and engineers and in this research, it serves as a basis for comparison between FSL network results and softmax-based one.

### 2.6. Metrics

Neural networks were evaluated using the metrics described below [16]. For simplicity of equations, specific acronyms have been created, as follows: TP—True Positive, TN—True Negative, FP—False Positive, FN—False Negative. The metrics used for network evaluation are:

- Accuracy: Acc = (TP+TN)/(TP+FP+TN+FN);
- Precision=TP/(TP+FP);
- Recall=TP/(TP+FN);
- F1=2·Precision·Recall/(Precision+Recall);
- AUC—Area Under ROC. ROC (Receiver operating characteristic) is a curve determined by calculating the True Positive Rate = TFP=TP/(TP+FN) and the False Positive Rate = FPR=FP/(TN+FP). The False Positive Rate describes the *x*-axis and the True Positive Rate the *y*-axis of a coordinate system. By changing the threshold value responsible for the classification of an example as belonging to either the positive or negative class, pairs of TFP–FPR are generated, resulting in the creation of the ROC curve. AUC is a measurement of the area below the ROC curve.

## 3. Results

**Figure 6:**
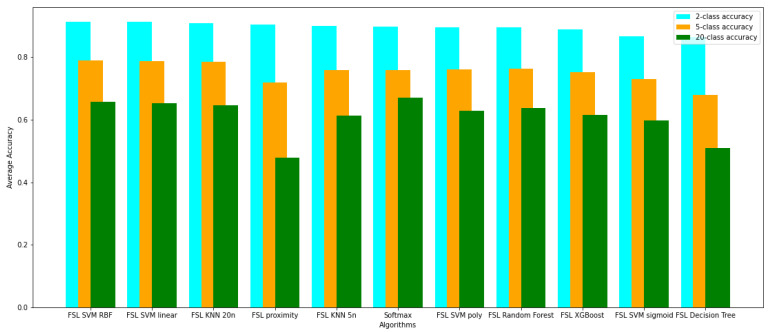
Comparison of average accuracy of evaluated models on 2, 5, and 20 classes detection.

**Figure 7:**
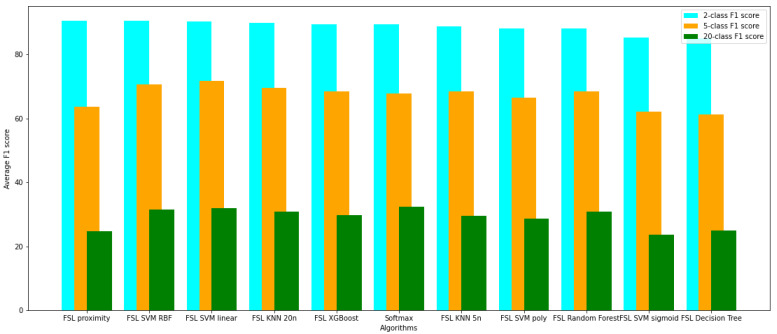
Comparison of average F1 score of evaluated models on 2, 5, and 20 classes detection.

The networks have been evaluated using the k-fold cross-validation technique for k=5. Each network has been trained five times from scratch on the randomly selected train, validation, and test datasets. The evaluation results on the test dataset are presented in Table 2, Table 3, Table 4, Table 5, Table 6 and Table 7 for tasks involving the classification of 2, 5, and 20 classes, respectively. Tables show the averaged, minimal, and maximal accuracy values and the F1, AUC, and specificity and sensitivity scores with standard deviation. Additionally, the average accuracy and the F1 score achieved by the evaluated models have been presented in Figure 6 and Figure 7.

**Figure 8:**
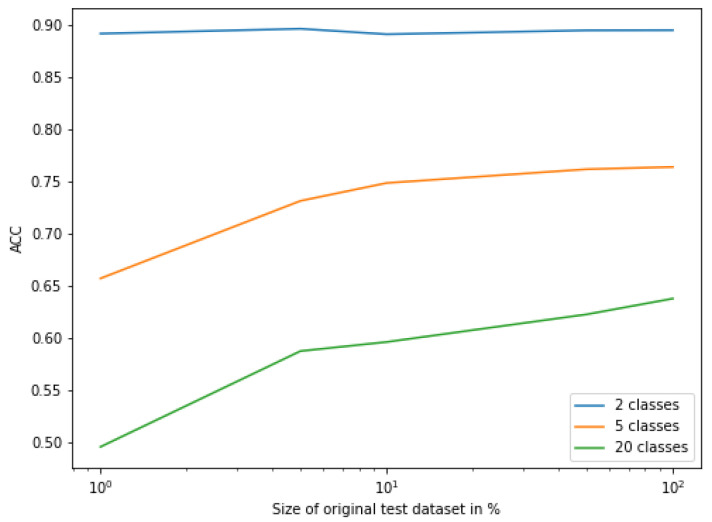
ACC as a function of the size of the original test dataset.

The influence of the dataset size on the FSL classification has been examined. During this evaluation, the Random Forest algorithm was used to classify few-shot encoded signals. The results are depicted in Figure 8, which shows the relationship between the size of the dataset used and the accuracy obtained during test evaluation. The sizes of the datasets evaluated are 1%, 5%, 10%, 50%, and 100% of the size of original test dataset.

Figure 9, Figure 10, Figure 11, Figure 12, Figure 13 and Figure 14 present the confusion matrices from the evaluation on one of the test datasets composed for k-fold cross validation conduction purposes. The Figure 15, Figure 16, Figure 17, Figure 18, Figure 19 and Figure 20 depict the accuracy on the training and validation datasets during the training process.

## 4. Discussion

The Deep Neural Network trained in a Few-Shot learning (FSL) fashion for proximity-based classification provides the benefit of improved accuracy through an embedded version of online learning, allowing for continuous classification augmentation without network weight adjustments. The network’s accuracy can be improved without the additional optimization of its weights through the expansion of the classified signals dataset. Such a set is used for referential class vector computation and is essential for the correct signal classification. Cardiological professionals can improve the network by labeling the signal and increasing the number of vectors used for class vector calculation, resulting in better classification. Such a procedure does not require training of the network, which is cumbersome on production machines due to the higher computation complexity of the training network than using an already trained one. This augmentation procedure can be conducted on a CPU with low computation capabilities due to the simplicity of mean vector calculation.

**Figure 11:**
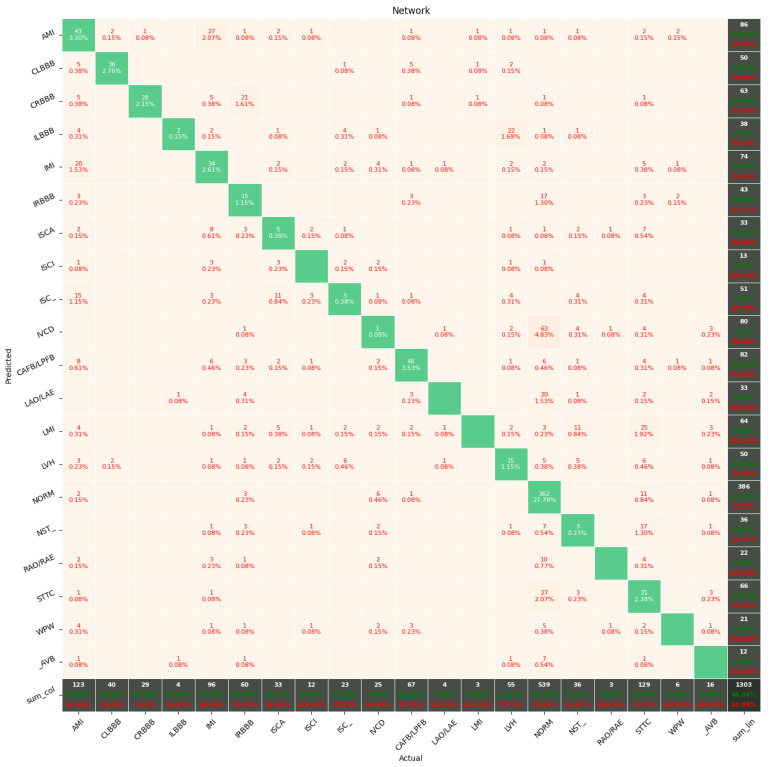
Confusion Matrix for Few-Shot (20 classes) with proximity-based classification.

The Few-Shot Learning neural network proved to be more accurate than the softmax-based network while classifying two classes. The FSL model had higher results in both averages, maximal and minimal accuracy. However, the network proved to be less accurate on tasks involving 5- and 20-class labeling. This phenomenon is most likely a result of insufficient representation of classes with low cardinality. For example: In Figure 11, the class “NORM” having the highest number of ECG records had the best precision and recall of all classes. The authors plan a further examination of the dataset size’s influence on the quality of prediction.

This work classified the signals processed by an FSL neural network by computing the average vector representing each class and comparing the Euclidean distance between the classified sample and all class-representing vectors. The other methods evaluated in this work for classification use network-encoded signals in small-sized vectors to train models running algorithms such as XGBoost, Random Forest, Decision Tree, K-Nearest Neighbors, and SVMs with linear, polynomial, radial basis function, and sigmoid kernels. It turned out that the most promising classification algorithm for FSL in this particular task is SVM with a radial basis function kernel. This method proved to be the most effective among all the examined FSL classification strategies and achieved better results than softmax-based classification for both two and five classes. It achieved one of the highest scores in accuracy, specificity, sensitivity, F1, and AUC among all compared models. The outcomes are promising and suggest that the hybrid neural network systems based on proximity-differentiation classification with integrated machine learning models may provide better results than the typical softmax-based state-of-the-art classification. The authors plan on conducting further research to determine whether a combination of FSL with SVM with radial basis function kernel is beneficial in other tasks or merely the case in this particular example.

**Figure 15:**
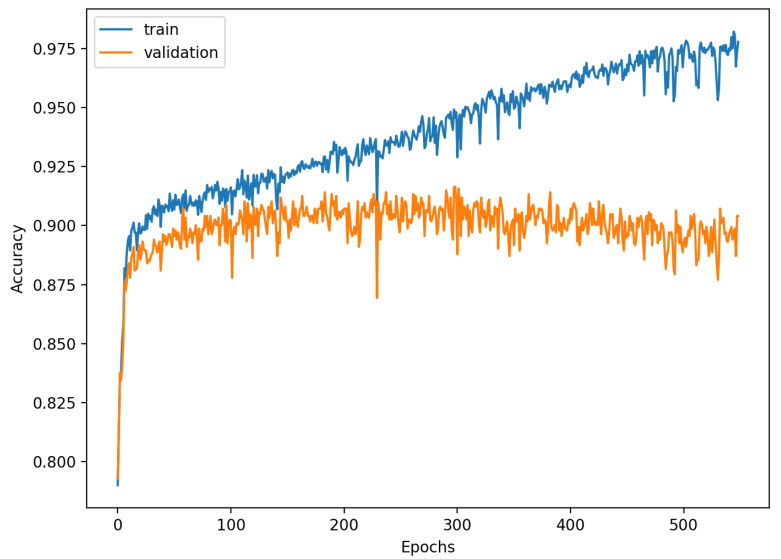
Learning process of the Neural Network for Few-Shot (2 classes) with proximity-based classification.

**Figure 16:**
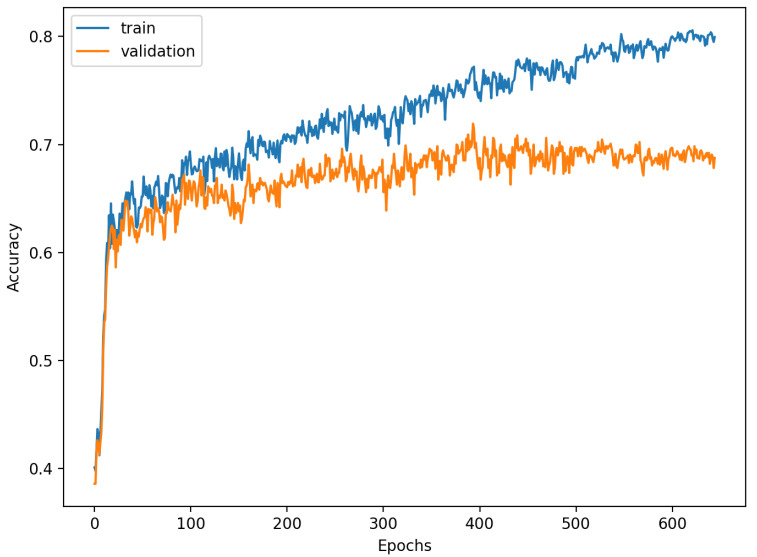
Learning process of the Neural Network for Few-Shot (5 classes) with proximity-based classification.

**Figure 17:**
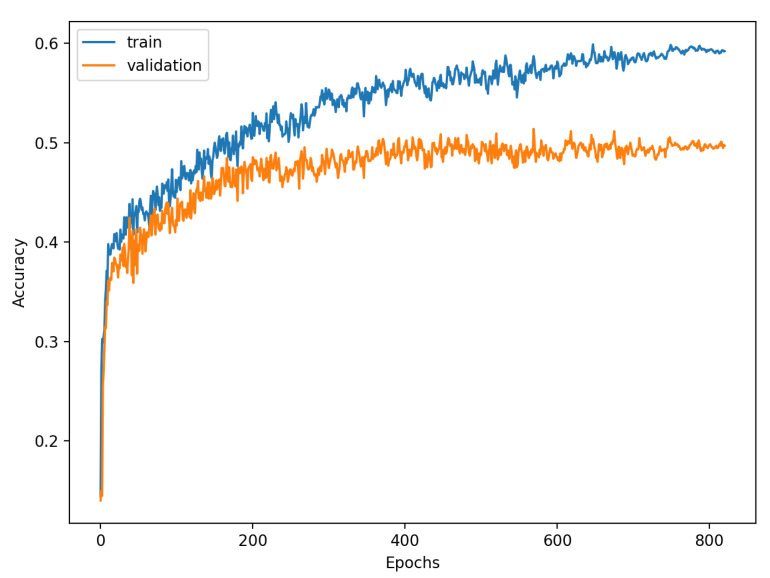
Learning process of the Neural Network for Few-Shot (20 classes) with proximity-based classification.

**Figure 18:**
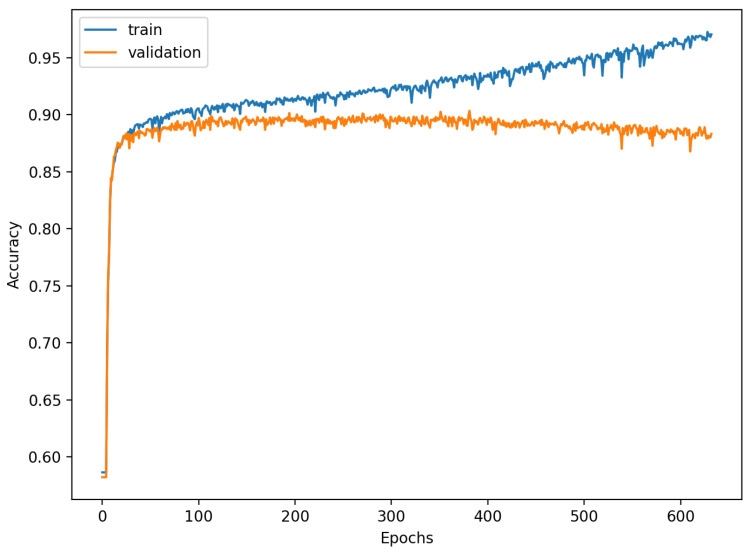
Learning process of the Neural Network for softmax-based classification (2 classes).

**Figure 19:**
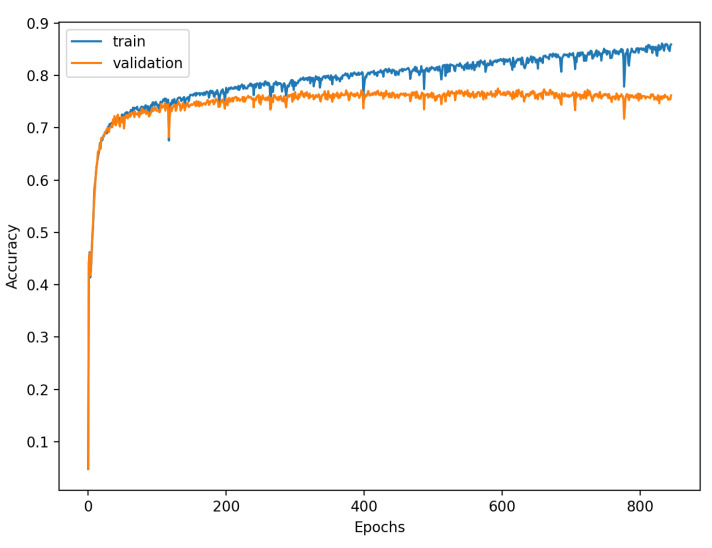
Learning process of the Neural Network for softmax-based classification (5 classes).

**Figure 20:**
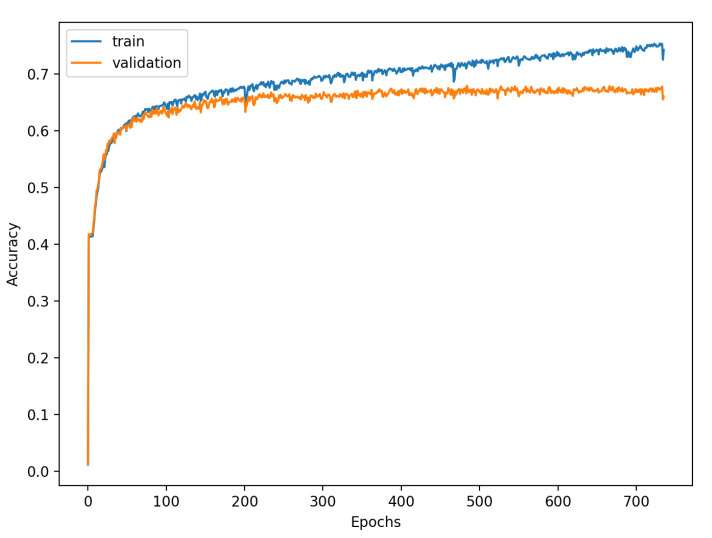
Learning process of the Neural Network for softmax-based classification (20 classes).

The accuracy of the FSL network during the training process varies significantly more than its softmax-based counterpart. This phenomenon is depicted in Figure 15, Figure 16, Figure 17, Figure 18, Figure 19 and Figure 20. The softmax-based classification network reaches convergence faster and is less susceptible to the noise generated by the random selection of training data. This variance of the learning process is essential because of the commonness of early-stopping usage during network training. Typical early-stopping implemented in DL frameworks such as Keras stops the training if the evaluation score of the trained network on the validation dataset was not improved in a specific amount of time. This mechanism is important as it reduces the amount of wasted computation time and energy. However, due to the high variance of the FSL process, it is possible that controlling early-stopping based on local extremum may not be the best strategy. The results indicate that filtration of evaluation score’s signal, such as averaging, may prove beneficial. The authors plan on further examination in future works.

In previous work [16], the best-obtained result in that research classifying sick/healthy patients (2 classes) is 89.2% accuracy. This value was increased in this research by the FSL neural network, the accuracy of which spans from 89.5% to 91.1%. As a result, even the worst performance of the studied network was better than the best in previous work. However, the results were not as promising during the classification of 5 and 20 classes. It is speculated that FSL can obtain better results for bigger datasets than Softmax-based classification, but the latter requires less training data than the former. The authors plan on conducting further research of this phenomenon.

The dataset size had almost no influence on the classification performance of the two classes. However, its impact was significant for the classification of 5 classes and even more important for the classification of 20 classes. It turns out that the more that classes are differentiated from each other, the more data are required.

## 5. Conclusions

The neural network trained for conducting Few-Shot Learning classification tasks proved to be more accurate than the softmax-based classification network when classifying signals using 2 and 5 labels but obtained worse results on 20 classes with fewer samples per class. In this experiment, the most efficient method for performing classification using the FSL network for signal encoding is the SVM model with an RBF kernel. Such networks can be successfully applied in systems that provide feedback from experts and data accumulation such as hospitals. The network can be improved without optimizing the network parameters in this environment, which requires high-end processing units such as GPUs. A proposed online learning strategy can be conducted on typical industrial CPUs. The FSL networks may prove beneficial as they allow for their performance to be improved after their rollout.

## Figures and Tables

**Figure 9:**
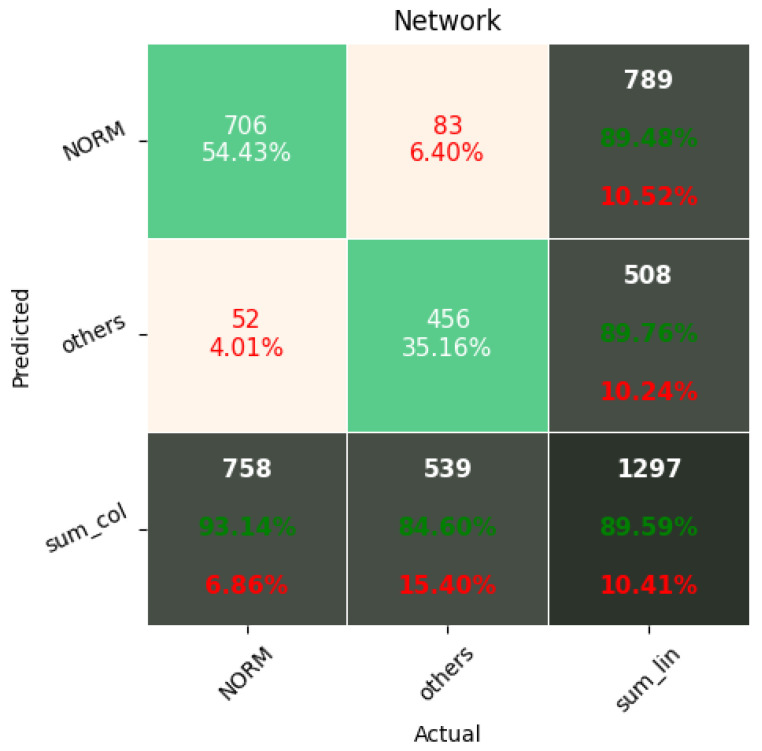
Confusion Matrix for Few-Shot (2 classes) with proximity-based classification.

**Figure 10:**
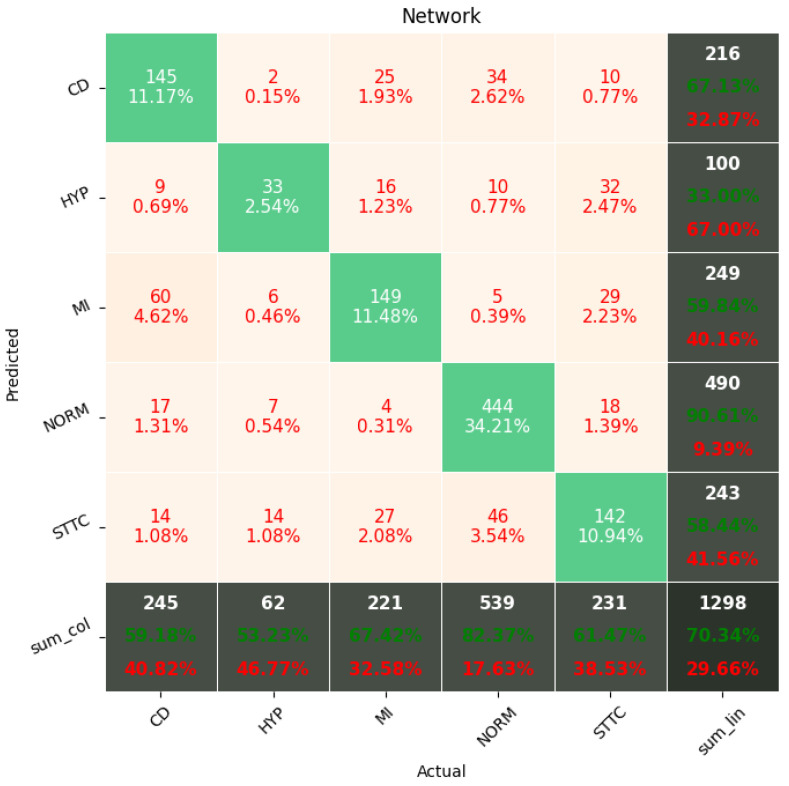
Confusion Matrix for Few-Shot (5 classes) with proximity-based classification.

**Figure 12:**
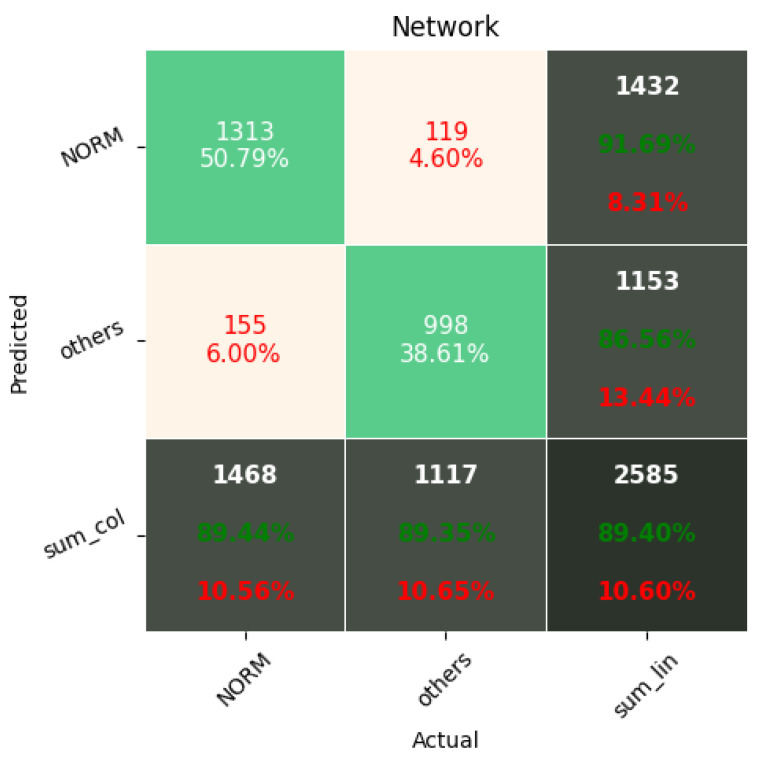
Confusion Matrix for softmax-based classification (2 classes).

**Figure 13:**
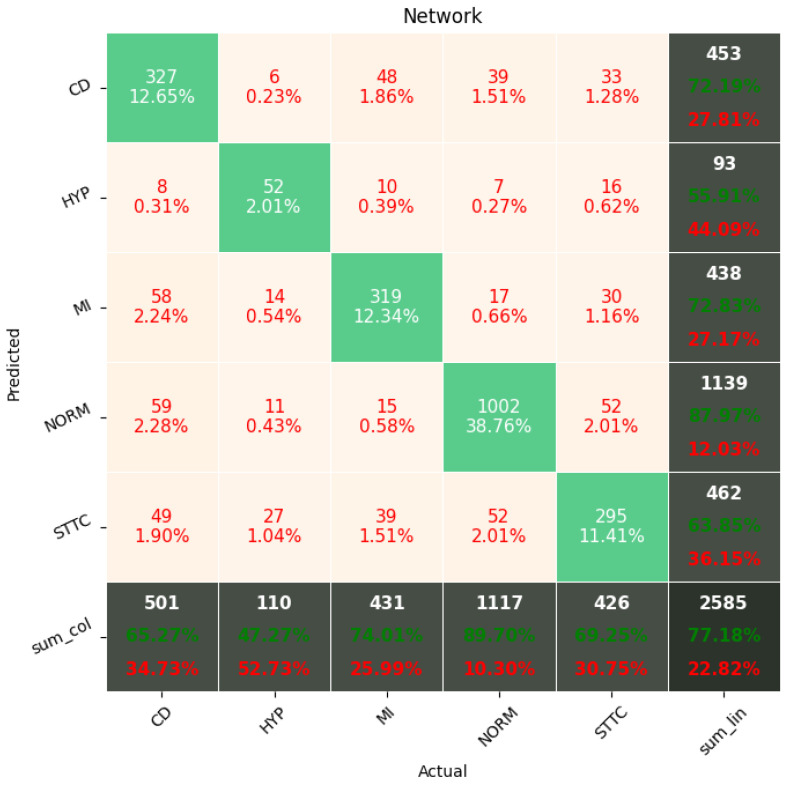
Confusion Matrix for softmax-based classification (5 classes).

**Figure 14:**
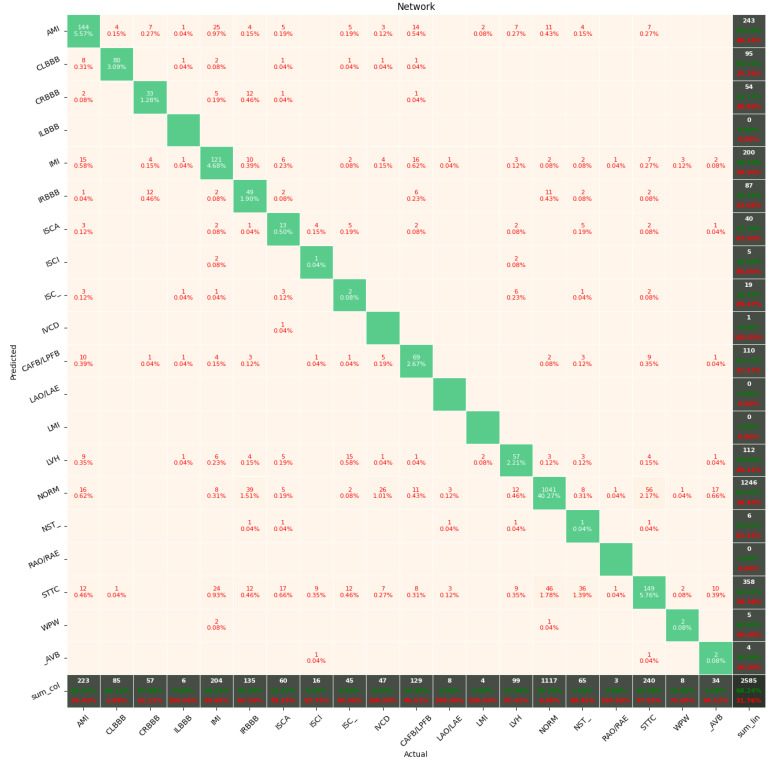
Confusion Matrix for softmax-based classification (20 classes).

**Table 1:** The architecture of Deep Convolutional Neural Network encoding one QRS complex.

Layer	Channels In	Channels Out	Kernel Size	Padding	Stride
Conv1d	12	24	3	1	1
MaxPool1d	24	24	2	0	2
Conv1d	24	48	3	0	1
MaxPool1d	48	48	2	0	2
Conv1d	48	96	3	0	1
MaxPool1d	96	96	2	0	2
Conv1d	96	2	1	0	1

**Table 2:** Results for two-class classification, part I.

Technique	Acc	Acc Avg | Std	F1	F1 Avg | Std	AUC	AUC Avg | Std
FSL proximity-based	89.5–91.1%	90.4% | 0.5%	89.1–90.8	90.6 | 0.6	92.5–94.4	93.7 | 0.8
Softmax-based classification	89.2–90.5%	89.7% | 0.4%	89.0–90.2	89.4 | 0.4	94.8–95.9	95.5 | 0.4
FSL + XGBoost	87.9–89.7%	88.9% | 0.7%	86.5–88.5	87.7 | 0.8	95.1–97.2	96.1 | 0.7
FSL + Random Forest	87.8–91.2%	89.4% | 1.1%	86.2–90.1	88.1 | 1.3	95.5–97.1	96.3 | 0.5
FSL + Decision Tree	84.9–88.9%	86.4% | 1.4%	82.8–87.5	85.0 | 1.8	82.8–87.5	85.0 | 1.8
FSL + KNN − 5 neighbors	88.7–92.0%	89.9% | 1.2%	87.1–91.2	88.8 | 1.4	93.9–96.4	94.6 | 0.9
FSL + KNN − 20 neighbors	88.1–93.3%	90.9% | 1.9%	86.6–92.6	89.8 | 2.2	96.0–97.8	96.6 | 0.7
FSL + SVM with linear kernel	88.6–93.3%	91.2% | 1.6%	87.4–92.9	90.3 | 1.8	96.1–97.6	96.9 | 0.5
FSL + SVM with polynomial kernel	87.2–93.0%	89.6% | 1.9%	85.5–92.3	88.2 | 2.3	94.9–97.6	96.0 | 1.0
FSL + SVM with RBF kernel	89.2–93.3%	91.3% | 1.4%	88.1–92.8	90.5 | 1.6	92.2–95.6	93.8 | 1.3
FSL + SVM with Sigmoid kernel	68.6–92.9%	86.6% | 9.1%	66.2–92.2	85.3 | 9.6	83.6–95.3	88.2 | 4.0

**Table 3:** Results for two-class classification, part II.

Technique	Specificity	Specificity Avg | Std	Sensitivity	Sensitivity Avg | Std
FSL proximity-based	89.6–91.0%	90.4% | 0.5%	88.9–90.7%	89.9% | 0.6%
Softmax-based classification	89.0–90.2%	89.4% | 0.5%	88.9–90.7%	89.9% | 0.6%
FSL + XGBoost	89.1–90.7%	89.8% | 0.6%	86.5–88.5%	87.7% | 0.8%
FSL + Random Forest	89.4–91.9%	90.3% | 1.0%	86.3–90.1%	88.2% | 1.3%
FSL + Decision Tree	86.4–89.7%	87.6% | 1.2%	82.8–87.5%	85.0% | 1.8%
FSL + KNN − 5 neighbors	89.6–92.7%	90.8% | 1.1%	87.1–91.2%	88.8% | 1.4%
FSL + KNN − 20 neighbors	89.4–93.9%	91.6% | 1.7%	86.6–92.6%	89.8% | 2.2%
FSL + SVM with linear kernel	89.5–93.5%	91.6% | 1.5%	87.4–92.9%	90.3% | 1.8%
FSL + SVM with polynomial kernel	89.2–93.7%	91.0% | 1.6%	85.5–92.3%	88.2% | 2.3%
FSL + SVM with RBF kernel	90.0–93.5%	91.7% | 1.3%	88.1–92.8%	90.5% | 1.6%
FSL + SVM with Sigmoid kernel	68.2–93.4%	87.2% | 9.6%	66.2–92.2%	85.3% | 9.6%

**Table 4:** Results for five-class classification, part I.

Technique	Acc	Acc Avg | Std	F1	F1 Avg | Std	AUC	AUC Avg | Std
FSL proximity-based	69.8–74.2%	71.8% | 1.7%	60.6–66.9	63.7 | 2.4	85.6–88.9	87.6 | 1.2
Softmax-based classification	75.1–77.1%	75.8% | 0.8%	66.8–69.6	67.9 | 1.0	87.5–90.9	89.6 | 1.3
FSL + XGBoost	74.8–76.1%	75.2% | 0.5%	66.9–70.8	68.4 | 1.6	90.9–92.3	91.8 | 0.5
FSL + Random Forest	75.2–77.7%	76.3% | 0.8%	66.8–69.9	68.4 | 1.1	92.0–93.0	92.5 | 0.4
FSL + Decision Tree	67.0–68.5%	68.0% | 0.5%	58.7–63.3	61.3 | 1.4	75.2–77.8	76.7 | 0.8
FSL + KNN − 5 neighbors	74.4–76.7%	75.8% | 0.8%	65.9–71.1	68.4 | 2.1	87.5–89.8	88.8 | 0.8
FSL + KNN − 20 neighbors	77.3–79.5%	78.4% | 0.9%	68.2–71.6	69.6 | 1.2	92.0–93.2	92.5 | 0.5
FSL + SVM with linear kernel	77.0–79.8%	78.8% | 1.0%	69.9–73.1	71.7 | 1.1	93.4–94.3	93.8 | 0.3
FSL + SVM with polynomial kernel	74.5–76.9%	76.0% | 0.9%	64.7–69.2	66.6 | 1.6	92.4–93.2	92.9 | 0.3
FSL + SVM with RBF kernel	77.9–80.2%	79.0% | 0.9%	69.0–71.8	70.6 | 1.0	93.3–93.8	93.6 | 0.3
FSL + SVM with Sigmoid kernel	64.4–76.6%	72.9% | 4.4%	53.1–65.0	62.1 | 4.5	89.5–92.8	90.8 | 1.1

**Table 5:** Results for five-class classification, part II.

Technique	Specificity	Specificity Avg | Std	Sensitivity	Sensitivity Avg | Std
FSL proximity-based	60.2–66.4%	63.2% | 2.1%	62.7–68.1%	65.9% | 2.0%
Softmax-based classification	68.3–70.6%	69.5% | 0.8%	65.9–69.1%	67.1% | 1.1%
FSL + XGBoost	65.7–68.0%	66.9% | 0.9%	66.9–70.8%	68.4% | 1.7%
FSL + Random Forest	66.8–68.3%	67.8% | 0.6%	66.8–69.9%	68.4% | 1.2%
FSL + Decision Tree	57.1–59.9%	59.0% | 1.0%	58.8–63.4%	61.3% | 1.5%
FSL + KNN − 5 neighbors	64.9–70.0%	67.6% | 1.8%	65.9–71.1%	68.4% | 2.1%
FSL + KNN − 20 neighbors	70.2–72.9%	71.9% | 1.0%	68.2–71.6%	69.6% | 1.2%
FSL + SVM with linear kernel	69.8–74.5%	72.4% | 1.6%	69.9–73.1%	71.7% | 1.1%
FSL + SVM with polynomial kernel	68.3–75.5%	72.4% | 2.5%	64.7–69.2%	66.6% | 1.6%
FSL + SVM with RBF kernel	70.8–75.5%	73.5% | 1.8%	69.0–71.8%	70.6% | 1.0%
FSL + SVM with Sigmoid kernel	56.1–70.8%	65.4% | 5.1%	53.1–65.0%	62.1% | 4.5%

**Table 6:** Results for 20-class classification, part I.

Technique	Acc	Acc Avg | Std	F1	F1 Avg | Std	AUC	AUC Avg | Std
FSL proximity-based	44.3–50.1%	47.8% | 2.1%	23.8–26.0	24.7 | 0.8	78.8–84.4	80.8 | 2.5
Softmax-based classification	66.2–68.2%	67.1% | 0.8%	31.9–33.0	32.4 | 0.4	82.4–86.3	84.4 | 1.5
FSL + XGBoost	58.7–66.2%	61.6% | 0.5%	25.3–34.5	29.7 | 3.4	74.2–86.3	79.3 | 3.5
FSL + Random Forest	61.5–66.6%	63.6% | 2.5%	27.1–36.7	30.9 | 3.5	73.6–80.3	77.1 | 2.3
FSL + Decision Tree	45.8–58.8%	51.0% | 4.5%	19.8–30.0	25.0 | 4.1	58.4–60.3	59.5 | 0.6
FSL +KNN − 5 neighbors	58.4–67.2%	61.3% | 3.2%	25.3–36.1	29.6 | 4.1	66.1–70.2	68.2 | 1.6
FSL + KNN − 20 neighbors	61.4–69.9%	64.6% | 2.9%	26.7–36.5	30.9 | 4.0	70.1–76.6	74.1 | 2.3
FSL + SVM with linear kernel	62.9–70.0%	65.3% | 2.5%	28.2–36.7	32.0 | 3.2	77.7–85.8	82.7 | 3.0
FSL + SVM with polynomial kernel	59.7–67.2%	62.9% | 2.8%	23.4–34.0	28.7 | 4.3	74.7–84.9	80.1 | 3.7
FSL + SVM with RBF kernel	63.3–70.6%	65.8% | 2.5%	27.8–37.2	31.4 | 3.9	77.1–82.5	80.5 | 2.2
FSL + SVM with Sigmoid kernel	56.7–65.4%	59.8% | 3.2%	16.6–28.2	23.7 | 4.6	77.0–82.5	80.9 | 2.0

**Table 7:** Results for 20-class classification, part II.

Technique	Specificity	Specificity Avg | Std	Sensitivity	Sensitivity Avg | Std
FSL proximity-based	24.9–26.8%	25.6% | 0.6%	27.7–29.7%	28.5% | 0.7%
Softmax-based classification	36.3–39.7%	37.6% | 1.2%	32.2–33.1%	32.6% | 0.3%
FSL + XGBoost	23.8–31.6%	27.7% | 2.6%	25.3–34.5%	29.7% | 3.4%
FSL + Random Forest	26.6–32.3%	28.4% | 2.2%	27.1–36.7%	31.0% | 3.6%
FSL + Decision Tree	19.4–28.5%	24.8% | 3.7%	19.9–30.0%	25.1% | 4.2%
FSL +KNN − 5 neighbors	24.0–33.7%	28.3% | 3.3%	25.3–36.1%	29.6% | 4.1%
FSL + KNN − 20 neighbors	24.9–31.1%	28.2% | 2.0%	26.7–36.5%	30.9% | 4.0%
FSL + SVM with linear kernel	25.6–34.1%	28.8% | 3.1%	28.2–36.7%	32.0% | 3.2%
FSL + SVM with polynomial kernel	26.0–33.0%	29.1% | 2.4%	23.4–34.0%	28.7% | 4.3%
FSL + SVM with RBF kernel	25.5–31.1%	28.9% | 2.7%	27.8–37.2%	31.4% | 3.9%
FSL + SVM with Sigmoid kernel	15.5–25.4%	20.8% | 3.4%	16.6–28.2%	23.7% | 4.6%

## Data Availability

The data presented in this study are available on request from the corresponding author.

## Nomenclature

ECG	Electrocardiogram
EEG	Electroencephalography
CT	Computed Tomography
QRS complex	Combination of three of the graphical deflections (Q wave, R wave, and S wave) seen on a typical ECG record. It represents an electrical impulse spreading through the ventricles of the heart and indicating their depolarization
Conv1d	Layer in Deep Neural Networks that performs a convolution on one-dimensional signal
MaxPool1d	Layer in Deep Neural Networks that performs pooling operation by selecting a maximum value from the moving window
Fully Connected	Layer in Deep Neural Networks that consists of neurons that process whole input data
Leaky ReLU	Activation function used in Deep Neural Networks
Padding	Parameter used in convolutional layers specifying the amount of zeroed samples added to the start and end of the processed signal. For example: Padding of 1 means that there is one sample of value zero artificially added at the beginning and the end of the signal. This operation is conducted to mitigate activation map shrinkage due to the application of convolution
Stride	Parameter used in convolutional layers specifying shift distance between subsequent windows of convolutions. For example: A stride of 1 means that the next convolution starts right after the beginning of the previous one, so the windows will overlap (provided that kernel size is bigger than 1)
RBF	Radial Basis Function

## Appendix A. Descriptions of the Classes of Diseases

NORM	Normal ECG
CD	Myocardial Infarction
STTC	ST/T Change
MI	Conduction Disturbance
HYP	Hypertrophy

## Appendix B. Descriptions of the Subclasses of Diseases

NORM	normal ECG
STTC	non-diagnostic T abnormalities, suggests digitalis-effect, long QT-interval, ST-T changes compatible with ventricular aneurysm, compatible with electrolyte abnormalities
AMI	anterior myocardial infarction, anterolateral myocardial infarction, in anteroseptal leads, in anterolateral leads, in lateral leads
IMI	inferior myocardial infarction, inferolateral myocardial infarction, inferoposterolateral myocardial infarction, inferoposterior myocardial infarction, in inferior leads, in inferolateral leads
LAFB/LPFB	left anterior fascicular block, left posterior fascicular block
IRBBB	incomplete right bundle branch block
LVH	left ventricular hypertrophy
CLBBB	(complete) left bundle branch block
NST_	non-specific ST changes
ISCA	in anterolateral leads, in anteroseptal leads, in lateral leads, in anterior leads
CRBBB	(complete) right bundle branch block
IVCD	non-specific intraventricular conduction disturbance
ISC_	ischemic ST-T changes
_AVB	first degree AV block, second degree AV block, third degree AV block
ISCI	in inferior leads, in inferolateral leads
